# From bouts to readiness to compete: temporal dynamics of stress, fatigue, recovery and monitoring-informed decisions in combat sports

**DOI:** 10.3389/fspor.2026.1969059

**Published:** 2026-10-29

**Authors:** Alex Ojeda-Aravena

**Affiliations:** 1 Departamento de Ciencias de la Actividad Física, Universidad de Los Lagos, Osorno, Chile; 2 School of Kinesiology, Universidad Bernardo O’Higgins, Santiago, Chile

**Keywords:** athletes, martial arts, decision making, fatigue, athletic performance, physical exertion

## Abstract

Combat-sport tournaments require decisions before responses to previous efforts have resolved. Detecting a change does not establish that an indicator predicts the next task or improves a decision. This Perspective proposes a task- and time-specific framework linking prior bouts, recovery time and subsequent demands through three evidentiary requirements. Responsiveness concerns reproducible changes beyond measurement error when an indicator represents a response to effort. Predictive validity concerns anticipation of a pre-specified subsequent outcome and added information beyond simpler measures. Decision utility concerns improved outcomes relative to usual practice. Indicators interpreted as current status or stable capacity need not respond to prior effort, but still require adequate measurement properties and prospective evaluation. Evidence from repeated bouts, recovery interventions, mental fatigue, sleep restriction and weight loss suggests that monitoring domains are not interchangeable. The framework retains discordant indicators, evaluates combinations and communicates uncertainty without global clearance labels. Three hypotheses address task and time dependence, maintained performance with increasing effort cost, and indicator-informed decisions. Medical decisions remain independent and take precedence. This is an operational extension of existing principles, not a validated predictive tool.

## 1 Introduction

Combat sports couple physiological and neuromuscular demands with perceptual-cognitive processing, psychological stress and tactical adaptation to an opponent. Athletes must sustain force and repeated actions while anticipating, selecting or inhibiting responses and adjusting distance or grips (1–4). Competitive anxiety and autonomic responses further contextualize performance (5). Because these demands interact under rules, scoring, contact and bout structures that differ across judo, wrestling, boxing, taekwondo and karate, preservation of a single capacity cannot establish readiness for the next bout.

These interacting demands highlight a practical gap in translating current information into the next decision. Perceived fatigue and performance fatigability are distinct (6); neither alone establishes capacity for another task later. This Perspective concerns the next bout during a tournament and demanding training or competition afterward. Readiness is defined here as an uncertain, prospective inference about whether the athlete's current state is sufficient for a specific task at a specific time, considering prior bouts and recovery time, rather than a global recovered/not-recovered state.

Competition studies illustrate this distinction. In official judo competition, perceived exertion increased from the first bout, whereas handgrip strength decreased from the third (7). In taekwondo, post-bout lactate and heart rate did not distinguish winners from non-winners (1). These findings illustrate the difference between detecting a response, describing the completed bout and predicting subsequent performance. These findings neither establish individual thresholds nor rule out the value of these measures for other outcomes.

Monitoring means repeated collection of load, physiological, perceptual, contextual and functional information to support decisions (8, 9). Health surveillance is complementary and requires independent clinical assessment. Existing approaches distinguish external work from internal response (10, 11), integrate load, status and response (12), and connect interpretation to action (9). Autonomic indices and wearable-derived measures require standardization and temporal and individual context (13–16). The contribution here is to organize these principles around competitive transitions and three evidentiary requirements (Table 1).

**Table 1:** Contribution of the present framework relative to existing athlete-monitoring approaches.

Approach	Main contribution	Tournament question	Proposed implication
Training-load monitoring (8, 10, 11)	Separates external work and internal response	What load accumulated?	High load alone does not justify reducing the next demand.
Monitoring cycle and decision support (9, 17, 18)	Connects data with action	Which action, and when?	Specify action, comparator and subsequent outcome before using a flag.
Fatigue and recovery frameworks (6, 19, 20)	Treats recovery as multidimensional	Which domains changed?	Retain discordance; reassess the function required next.
Multidimensional monitoring of training effects (12)	Integrates load, status and response	Does added information predict the next task?	Keep additional measures only when they improve prediction and decisions.
Distinction between immediate readiness and long-term preparedness (21)	Separates immediate state and long-term capacity	What is sufficient for this task?	Distinguish reversible adjustments from routine restriction.

Against this background, the objective of this Perspective is to propose a task- and time-specific framework specifying the evidence needed for monitoring-informed decisions between and after combat-sport bouts. It addresses researchers and applied sport scientists, with implications for coaches. Long-term adaptation planning lies outside its scope, and no universal participation threshold is proposed; prospective validation is required. The cited studies were selected to illustrate limitations in inference, not to estimate effects systematically. Medical restrictions take precedence, particularly after concussion (22). Findings in former professional boxers illustrate why preserved test performance cannot establish absence of neurological risk (23).

## 2 From precompetitive activation to post-bout responses

Interpretation begins before the bout because competitive activation can shape the measured response. Judo studies report anxiety, cortisol and heart rate variability (HRV) changes (5, 24). In youth taekwondo, alpha-amylase peaked at combat completion and cortisol later in recovery (25, 26). These findings contextualize measurements without establishing subsequent incapacity.

Experimental work adds mechanistic insight. A mixed martial arts training protocol reduced maximal force predominantly through peripheral fatigue, with possible central compensation (27), but this mechanism leaves open whether compensation persists through the next official bout. Mechanistic plausibility, moreover, cannot substitute for repeatability, prospective prediction or demonstrated benefit from acting on an indicator. At the level of task performance, fatiguing taekwondo tasks altered kick impact and reaction time unevenly (28); an upper-body Wingate altered reaction time and consistency in mixed martial arts athletes (29). These experiments support component-specific assessment, not validated tournament readiness classification.

Reproducing bout activity does not guarantee equivalent internal demands. Championship taekwondo produced greater physiological responses than an activity-matched protocol (30); official and simulated karate also differed (31). Controlled protocols remain useful for investigating mechanisms, but transfer to competitive decisions requires testing rather than assuming slower recovery or decision utility.

## 3 Interpreting differences between indicators during recovery

The same caution applies to recovery. Because fatigue is task-dependent and multidimensional (20), interpreting recovery requires specifying which function is measured, when, and against what reference.

### 3.1 Training and repeated-bout evidence

Over four weeks, young judokas showed differing trajectories in perceived recovery, HRV, grip and jump performance (32). Brazilian jiu-jitsu training altered perceptual and endocrine measures without significant jump changes (33). These findings describe training responses; they do not establish recovery trajectories during tournaments or isolate independent mechanisms.

Repeated-bout responses vary by protocol and metric (34). Simulated wrestling after weight loss produced progressive performance decline (35); female judo and repeated taekwondo bouts altered force, acid–base balance or physiological responses (36, 37), and successive simulated judo matches reduced shoulder torque and jump height (38). In judo, jump impulse changed while height and power did not (39). Wrestlers had not returned to pre-match HRV and jump values after brief recovery interventions (40). Together, these studies point to protocol-specific recovery rather than a universal interval. When weight loss and successive bouts occur together, their contributions cannot be separated from the time course alone; exposure history must remain part of interpretation.

Perceived and measured recovery can also diverge. Elite judokas rated compression garments favorably without corresponding performance or muscle-damage benefits (41). This contrast distinguishes perceived intervention effectiveness from recovery of capacity; a combined score would obscure rather than resolve the discrepancy.

### 3.2 Perceptual-cognitive functions

The picture extends beyond physical measures. Mental fatigue can impair sport-specific psychomotor performance (42), including temporal prediction in boxing (43). Case-based mixed martial arts findings further illustrate individual cognitive responses (44). Physiological measures within the usual range do not necessarily indicate preserved perceptual-cognitive function. Yet cognitive changes need not transfer to every sporting task. A modified Stroop task altered cognitive and neuroelectric responses in 15 taekwondo athletes without impairing the measured sport-specific visuomotor performance (45). Candidate tests therefore require familiarization and assessment of speed, accuracy, inhibition and added predictive value.

### 3.3 Context as part of indicator evaluation

Prior conditions also matter. Rapid weight loss affected hydration and judo performance (46, 47). Reviews show heterogeneous performance effects, with more consistent adverse mood and fatigue findings (48–50). Interpretation requires weight-loss method, magnitude and recovery interval (51). Sleep also contextualizes recovery (52). Four versus eight hours in bed impaired cognitive and physical outcomes in 14 male karate athletes, although sleep duration was not measured objectively (53). Sleep opportunity is contextual information, not a validated score correction.

Taken together, these studies show that recovery is multidimensional, yet much of the literature is intervention-focused (19, 54). Whether an indicator normalizes or remains altered, its relevance to subsequent capacity must be tested rather than assumed. Divergence motivates domain-specific assessment, not the assumption that more domains improve decisions.

## 4 Readiness for the next task during and after the tournament

The question therefore shifts from whether an indicator has returned to baseline to whether current information anticipates the next task. An athlete may tolerate technical training but not demanding contact, and a grip deviation may matter before another judo bout yet little before mobility work, a difference that itself requires testing. Building on immediate-state monitoring (12), the framework proposes that readiness interpretation should depend on subsequent demands rather than summed normalized indicators.

Task dependence also changes the consequences of error. Unnecessary restriction can remove useful training, whereas excessive demand can compromise performance; prioritizing daily readiness may displace long-term preparation (21). Thresholds should reflect both consequences (17, 55). The same error matters differently before an unavoidable bout than before a modifiable session. Justifying these inferences requires three distinct forms of evidence.

## 5 Three evidentiary requirements for using an indicator in decision-making

### 5.1 Responsiveness

The first requirement, responsiveness, asks whether an effort-related measure changes reproducibly beyond error and habitual variation (56, 57). A significant group change does not imply an unusual individual change, and interpretation must also consider timing, exposure and decision-relevant magnitude. High reliability is necessary but not sufficient. Judo jump metrics, for example, were reliable, yet only impulse changed across repeated bouts (39). This requirement depends on interpretation rather than test category, since grip may represent absolute capacity or post-bout change. All predictors require known measurement error and prospective validity; effort-response interpretations additionally require reproducible change.

### 5.2 Predictive validity

The second requirement, predictive validity, asks whether a measure anticipates a pre-specified outcome at a declared horizon, such as technical accuracy in the next bout or completion of demanding training. Bout outcome is a weaker validation target because it also depends on the opponent. HRV sensitivity during judo training, for example, is distinct from next-bout prediction (58). None of the studies reviewed here prospectively linked between-bout measurements to next-bout outcomes, although this reflects the scope of the present synthesis rather than proof that such evidence is absent.

Incremental validity requires comparison of a pre-specified model using documented coach-available information with one adding the indicator. Calibration, discrimination or continuous prediction error should account for repeated athletes and be evaluated in new tournaments (59–61). Poor external validation in sports injury research reinforces the distinction between associations and useful predictions (62). Comparison with an information-free benchmark would exaggerate added value. Because correlated predictors may add little beyond existing information (63), multiple fatigue measures should not be counted as independent confirmations. Self-report offers a simpler comparator (64); combinations must outperform a feasible alternative.

### 5.3 Decision utility

The third requirement concerns what happens when information is used. Decision utility balances performance, excessive demands, restrictions, burden and adherence against usual practice. Accurate prediction may change no action or produce harmful restrictions. Individualized caffeine dosing improved simulated wrestling performance (65), but an intervention effect does not validate this framework. Utility requires evaluation of implemented decisions and athlete-relevant outcomes. Tournament intervals constrain available actions; post-competition training offers greater scope for adjustments.

## 6 Translating monitoring into decisions

In practice, the next task and its timing guide selection of a small standardized set (66). Individual interpretation requires separating within-athlete from between-athlete variation (67), because individual reference values do not eliminate measurement error. These references draw on familiarized, comparable observations, with equipment, timing, exercise, sleep and weight-management context recorded. The required precision determines the number of observations, while detectable change remains distinct from decision relevance (56, 57). Since rested-training references may not transfer between bouts, personalized thresholds must communicate rather than conceal uncertainty.

Timing also determines what can be measured. Weigh-in timing, accumulated bouts and recovery intervals determine when information can inform the next decision, rather than when assessment is merely convenient. With minutes available, brief reporting, observation and a familiar low-burden test may be feasible; assessment must not displace recovery or preparation. Over days, grip, jump or perceptual-cognitive testing may be standardized. Grappling and striking likely require different outcomes; mixed demands do not automatically justify more tests. These are candidate configurations, not validated batteries.

Implementation also depends on shared interpretation. Researchers establish validity, scientists interpret and coaches adjust tasks (68, 69), incorporating athlete experience (70). Punitive self-report use can encourage concealment (71). Athletes should understand data access and purpose, and discuss options ranging from preparation adjustments to modified volume, intensity or contact. Monitoring informs these choices; it neither authorizes participation nor overrides medical restrictions. Within this dialogue, discordant domains remain informative because an unchanged jump does not cancel altered grip, and poor perceived recovery does not by itself indicate inability. Reversible adjustments permit reassessment. Communication should explain the finding, predictive limits, options and review time.

A hypothetical judoka illustrates the distinction. With 20 min before another bout, grip is 12% below an individual reference. Even if this exceeds measurement uncertainty, it does not prove next-bout impairment. Rather than triggering automatic restriction, the finding should be documented together with its uncertainty, any familiar preparation adjustment and the subsequent outcome, without presuming benefit. Symptoms trigger independent medical assessment. After competition, discordance likewise prompts task-specific reassessment rather than automatic cancellation of contact training.

## 7 Testable predictions

These inferential boundaries lead to three hypotheses about added value beyond simpler monitoring. Divergent trajectories motivate the framework, but retaining them must improve predictions or decisions. Meaningful effects require pre-specification from pilot precision and error consequences; individualization remains a comparator to evaluate rather than a presumed improvement.

First, task and time specification is hypothesized to improve prediction. A prospective study could measure grip across varying between-bout intervals and compare prediction of the same next-bout outcome with and without interval information. Evaluation before next-day training would test transfer to another task. Evaluation in held-out athletes or tournaments would assess calibration and discrimination for binary outcomes, or prediction error for continuous outcomes. Sufficiently precise evidence of no meaningful improvement would challenge the hypothesis. Within this design, deviations from individual references could be compared with absolute values and athlete characteristics. Reporting reference quality and uncertainty would distinguish individualization from noise.

Second, maintained performance with unusually high effort cost is hypothesized to predict subsequent decline. A candidate protocol would pair familiarized grip assessment with perceived effort during the same task at matched recovery times. Maintained output would be defined within measurement uncertainty, and elevated effort against comparable individual responses, using a pre-specified scale and anchors [e.g., Borg CR10 (72);]. A mixed-effects interaction model could be compared with main effects, controlling prior bouts and recovery time. Absence of added out-of-sample information would challenge this candidate pattern of compensation; its presence would not identify a mechanism.

Third, a specified non-medical rule is hypothesized to improve outcomes over usual practice. Randomized post-tournament periods or groups could evaluate a prospectively documented rule, accounting for repeated athletes and carry-over, with identical medical procedures. Outcomes would include session completion, performance, restrictions, adherence and harms, with trade-offs pre-specified. Decision curves cannot replace impact assessment (55). Sufficiently precise evidence of no benefit or worse outcomes would count against the hypothesis; staggered multiple-baseline designs could provide preliminary feasibility evidence when randomization is unavailable. Repeated failure of task/time specification and implemented rules would limit the framework's added value. Failure of a complex measure alone would instead favor simpler monitoring.

## 8 Discussion

The contribution is therefore not another battery or readiness score, but explicit inferential steps linking monitoring to competitive decisions. Existing approaches already integrate context (9, 12). Here, prior bouts, recovery time and the next task define the prediction and action to evaluate. Like Besson and Stucky (18), the framework distinguishes changing decisions from improving outcomes. Its added value requires improvement over simpler monitoring, including experienced coaches’ contextual judgment, which serves as a comparator rather than an approach to dismiss.

The evidence, however, supports caution more strongly than it validates a decision rule. Divergent judo responses and preserved sport-specific visuomotor performance despite cognitive changes suggest that domains are not interchangeable (7, 45). They do not show that retaining every domain improves prediction. Responsiveness does not imply predictive validity, and predictive validity does not imply decision utility, consistent with the progression in prognostic research (59, 60). Dynamical systems offer descriptive heuristics, not a necessary explanation or validated mechanism (73, 74).

Nevertheless, decisions cannot wait for complete validation. The aim is to expose uncertainty, not delay action. An unvalidated deviation may prompt reassessment, but not universal restriction. Familiar, reversible adjustments can be documented without presuming benefit. Measurement burden must be weighed against preparation and recovery; resource-limited implementation may record one brief measure, a decision and an outcome. Such records do not alone establish causality or justify overriding athlete preferences or medical restrictions.

Finally, the evidence base limits these claims. This targeted synthesis is not systematic; selection may favor the argument, and the examples cannot establish that prognostic studies are absent. Small samples and heterogeneous designs limit generalizability. Findings in high-level adults cannot automatically apply to younger or lower-level athletes, nor can grip represent striking accuracy. Validation must specify population, discipline, rules and available actions.

## 9 Conclusion

The practical question thus shifts from global recovery to what current information predicts for a defined task and whether acting on it improves outcomes. This favors fewer relevant indicators without erasing discordance or uncertainty. Progress is likely to depend less on recording more variables than on understanding when indicators change, what they anticipate and when that knowledge justifies acting differently. The next step is prospective competition research with specified outcomes and separate-tournament validation, followed by impact evaluation of a documented non-medical rule.

## Data Availability

The original contributions presented in the study are included in the article/Supplementary Material, further inquiries can be directed to the corresponding author.

## References

[1] Matsushigue KA Hartmann K Franchini E. Taekwondo: physiological responses and match analysis. J Strength Cond Res. (2009) 23(4):1112–7. 10.1519/JSC.0b013e3181a3c597 19528839

[2] Milazzo N Farrow D Ruffault A Fournier JF. Do karate fighters use situational probability information to improve decision-making performance during on-mat tasks? J Sports Sci. (2016) 34(16):1547–56. 10.1080/02640414.2015.1122824 26651505

[3] Bridge CA Ferreira Da Silva Santos J Chaabène H Pieter W & Franchini E. Physical and physiological profiles of taekwondo athletes. Sports Med. (2014) 44(6):713–33. 10.1007/s40279-014-0159-9 24549477

[4] Franchini E Del Vecchio FB Matsushigue KA Artioli GG. Physiological profiles of elite judo athletes. Sports Med. (2011) 41(2):147–66. 10.2165/11538580-000000000-00000 21244106

[5] Morales J Garcia V García-Massó X Salvá P Escobar R Buscà B. The use of heart rate variability in assessing precompetitive stress in high-standard judo athletes. Int J Sports Med. (2013) 34(2):144–51. 10.1055/s-0032-1323719 22972248

[6] Enoka RM Duchateau J. Translating fatigue to human performance. Med Sci Sports Exerc. (2016) 48(11):2228–38. 10.1249/MSS.0000000000000929 27015386 PMC5035715

[7] Kons RL Pupo JD Ache-Dias J Garcia T Da Silva RR Katicips LFG. Effect of official judo matches on handgrip strength and perceptual responses. J Exerc Rehabil. (2018) 14(1):93–9. 10.12965/jer.1835156.578 29511658 PMC5833974

[8] Bourdon PC Cardinale M Murray A Gastin P Kellmann M Varley MC. Monitoring athlete training loads: consensus statement. Int J Sports Physiol Perform. (2017) 12(Suppl. 2):S2-161–70. 10.1123/IJSPP.2017-0208 28463642

[9] Gabbett TJ Nassis GP Oetter E Pretorius J Johnston N Medina D. The athlete monitoring cycle: a practical guide to interpreting and applying training monitoring data. Br J Sports Med. (2017) 51(20):1451–2. 10.1136/bjsports-2016-097298 28646100

[10] Impellizzeri FM Marcora SM Coutts AJ. Internal and external training load: 15 years on. Int J Sports Physiol Perform. (2019) 14(2):270–3. 10.1123/ijspp.2018-0935 30614348

[11] Impellizzeri FM Shrier I McLaren SJ Coutts AJ McCall A Slattery K. Understanding training load as exposure and dose. Sports Med. (2023) 53(9):1667–79. 10.1007/s40279-023-01833-0 37022589 PMC10432367

[12] Rebelo A Bishop C Thorpe RT Turner AN Gabbett TJ. Monitoring training effects in athletes: a multidimensional framework for decision-making. Sports Med. (2026) 56(7):1603–24. 10.1007/s40279-026-02417-4 41824225 PMC13388359

[13] Bellenger CR Fuller JT Thomson RL Davison K Robertson EY Buckley JD. Monitoring athletic training status through autonomic heart rate regulation: a systematic review and meta-analysis. Sports Med. (2016) 46(10):1461–86. 10.1007/s40279-016-0484-2 26888648

[14] Buchheit M. Monitoring training status with HR measures: do all roads lead to Rome? Front Physiol. (2014) 5:73. 10.3389/fphys.2014.00073 24578692 PMC3936188

[15] Plews DJ Laursen PB Stanley J Kilding AE Buchheit M. Training adaptation and heart rate variability in elite endurance athletes: opening the door to effective monitoring. Sports Med. (2013) 43(9):773–81. 10.1007/s40279-013-0071-8 23852425

[16] Cardinale M Varley MC. Wearable training-monitoring technology: applications, challenges, and opportunities. Int J Sports Physiol Perform. (2017) 12(Suppl. 2):S2-55–62. 10.1123/ijspp.2016-0423 27834559

[17] Robertson S Bartlett JD Gastin PB. Red, amber, or green? Athlete monitoring in team sport: the need for decision-support systems. Int J Sports Physiol Perform. (2017) 12(Suppl. 2):S2-73–9. 10.1123/ijspp.2016-0541 27967289

[18] Besson C Stucky F. From monitoring metrics to decision evidence: validating actionability in applied sport science. Front Sports Act Living. (2026) 8:1983979. 10.3389/fspor.2026.1983979 42871857 PMC13661024

[19] Kellmann M Bertollo M Bosquet L Brink M Coutts AJ Duffield R. Recovery and performance in sport: consensus statement. Int J Sports Physiol Perform. (2018) 13(2):240–5. 10.1123/ijspp.2017-0759 29345524

[20] Behrens M Gube M Chaabene H Prieske O Zenon A Broscheid K-C. Fatigue and human performance: an updated framework. Sports Med. (2023) 53(1):7–31. 10.1007/s40279-022-01748-2 36258141 PMC9807493

[21] Kruczek K Rebelo A Gabbett T Nowak M. The readiness–preparedness bias: recalibrating monitoring logic. Front Physiol. (2026) 17:1855985. 10.3389/fphys.2026.1855985 42305917 PMC13265307

[22] Neidecker J Sethi NK Taylor R Monsell R Muzzi D Spizler B. Concussion management in combat sports: consensus statement from the association of ringside physicians. Br J Sports Med. (2019) 53(6):328–33. 10.1136/bjsports-2017-098799 30049779 PMC6579496

[23] Kamps S Van Amerongen S Blujdea ER Hoppen MI Bongers B Schelvis KH. Plasma and MRI biomarkers capture neuronal damage in former professional boxers. Sci Rep. (2025) 15(1):20005. 10.1038/s41598-025-93066-6 40481074 PMC12144257

[24] Filaire E Sagnol M Ferrand C Maso F Lac G. Psychophysiological stress in judo athletes during competitions. J Sports Med Phys Fitness. (2001) 41(2):263–8. Available online at: https://pubmed.ncbi.nlm.nih.gov/11447372/ (Accessed August 25, 2026). 11447372

[25] Capranica L Condello G Tornello F Iona T Chiodo S Valenzano A. Salivary alpha-amylase, salivary cortisol, and anxiety during a youth taekwondo championship: an observational study. Medicine (Baltimore). (2017) 96(28):e7272. 10.1097/MD.0000000000007272 28700470 PMC5515742

[26] Chiodo S Tessitore A Cortis C Cibelli G Lupo C Ammendolia A. Stress-related hormonal and psychological changes to official youth taekwondo competitions. Scand J Med Sci Sports. (2011) 21(1):111–9. 10.1111/j.1600-0838.2009.01046.x 20030779

[27] Giboin L-S Gruber M. Neuromuscular fatigue induced by a mixed martial art training protocol. J Strength Cond Res. (2022) 36(2):469–77. 10.1519/JSC.0000000000003468 31895287

[28] Sant'Ana J Franchini E Da Silva V Diefenthaeler F. Effect of fatigue on reaction time, response time, performance time, and kick impact in taekwondo roundhouse kick. Sports Biomechanics. (2017) 16(2):201–9. 10.1080/14763141.2016.1217347 27592682

[29] Pavelka R Třebický V Třebická Fialová J Zdobinský A Coufalová K Havlíček J. Acute fatigue affects reaction times and reaction consistency in mixed martial arts fighters. PLoS One. (2020) 15(1):e0227675. 10.1371/journal.pone.0227675 32004350 PMC6994193

[30] Bridge CA McNaughton LR Close GL Drust B. Taekwondo exercise protocols do not recreate the physiological responses of championship combat. Int J Sports Med. (2013) 34(7):573–81. 10.1055/s-0032-1327578 23296399

[31] Chaabène H Mkaouer B Franchini E Souissi N Selmi MA Nagra Y. Physiological responses and performance analysis difference between official and simulated karate combat conditions. Asian J Sports Med. (2014) 5(1):21–9. 10.5812/asjsm.34228 PMC4009084 24868428

[32] Campos BT Nakamura FY Penna EM Moraes RGSD Rodrigues JGDS Albuquerque MR. Monitoring training loads in judo athletes: different time courses of physiological, neuromuscular, and perceptual responses. Int J Exerc Sci. (2023) 16(6):638–53. 10.70252/HEAC5722 37621711 PMC10446951

[33] Campos F Molina Correa JC Canevari VCM Branco BHM Andreato LV De Paula Ramos S. Monitoring internal training load, stress-recovery responses, and immune-endocrine parameters in Brazilian jiu-jitsu training. J Strength Cond Res. (2022) 36(3):723–31. 10.1519/JSC.0000000000003507 31972820

[34] Kons RL Orssatto LBR Detanico D. Acute performance responses during repeated matches in combat sports: a systematic review. J Sci Med Sport. (2020) 23(5):512–8. 10.1016/j.jsams.2019.12.004 31843442

[35] Barbas I Fatouros IG Douroudos II Chatzinikolaou A Michailidis Y Draganidis D. Physiological and performance adaptations of elite greco-roman wrestlers during a one-day tournament. Eur J Appl Physiol. (2011) 111(7):1421–36. 10.1007/s00421-010-1761-7 21161266

[36] Bardin J Bourdier P Bontemps B Diry A Birat A Blazevich AJ. Repeated simulated match-induced changes in finger flexor force and blood acid-base balance in world-class female judokas. Int J Sports Physiol Perform. (2022) 17(10):1499–506. 10.1123/ijspp.2022-0057 35894825

[37] Bridge CA Sparks SA McNaughton LR Close GL Hausen M Gurgel J. Repeated exposure to taekwondo combat modulates the physiological and hormonal responses to subsequent bouts and recovery periods. J Strength Cond Res. (2018) 32(9):2529–41. 10.1519/JSC.0000000000002591 29781933

[38] Detanico D Pupo JD Franchini E Dos Santos SG. Effects of successive judo matches on fatigue and muscle damage markers. J Strength Cond Res. (2015) 29(4):1010–6. 10.1519/JSC.0000000000000746 25426512

[39] Kons RL Ache-Dias J Costa FE Dal Pupo J Detanico D. Effects of multiple judo bouts in the bilateral strength deficit on the countermovement jump metrics. J Hum Kinet. (2025) 100:17–26. 10.5114/jhk/202147 41766798 PMC12946876

[40] Güngör AK Topçu H Yildirim Y Flatt A. Effects of post-match foam rolling, static stretching, and passive rest on acute cardiac-autonomic, hemodynamic, and neuromuscular recovery in national wrestlers. BMC Sports Sci Med Rehabil. (2026) 18(1):145. 10.1186/s13102-026-01646-4 PMC13005558 41787568

[41] Brown FCW Hill JA Van Someren K Howatson G Pedlar CR. The effect of custom-fitted compression garments worn overnight for recovery from judo training in elite athletes. Eur J Sport Sci. (2022) 22(4):521–9. 10.1080/17461391.2021.1891294 33583335

[42] Habay J Van Cutsem J Verschueren J De Bock S Proost M De Wachter J. Mental fatigue and sport-specific psychomotor performance: a systematic review. Sports Med. (2021) 51(7):1527–48. 10.1007/s40279-021-01429-6 33710524

[43] Wu C-H Yang Y Xu X Wang N Li Q Geng L. Mental fatigue impairs temporal perceptual prediction: a study on boxing performance across skill levels. Sports. (2025) 13(5):154. 10.3390/sports13050154 40423290 PMC12115969

[44] Faro HK Fortes LS Machado DG. Dynamics of cognitive performance at rest and after exhaustive exercise in top-three world-ranked mixed martial arts athletes: a series of case studies. J Sports Med Phys Fitness. (2020) 60(4):664–8. 10.23736/S0022-4707.19.10207-1 31818054

[45] Faro H Franchini E Cavalcante-Silva D Da Silva RDM Barbosa BT Machado DGS. Do prolonged social media use or cognitive tasks impair neuroelectric and visuomotor performance in taekwondo athletes? A randomized and controlled trial. Psychol Sport Exerc. (2025) 76:102768. 10.1016/j.psychsport.2024.102768 39419239

[46] Ceylan B Aydos L Šimenko J. Effect of rapid weight loss on hydration status and performance in elite judo athletes. Biology (Basel). (2022) 11(4):500. 10.3390/biology11040500 35453700 PMC9031997

[47] Isacco L Degoutte F Ennequin G Pereira B Thivel D Filaire E. Rapid weight loss influences the physical, psychological and biological responses during a simulated competition in national judo athletes. Eur J Sport Sci. (2020) 20(5):580–91. 10.1080/17461391.2019.1657503 31418331

[48] Brechney GC Cannon J Goodman SP. Effects of weight cutting on exercise performance in combat athletes: a meta-analysis. Int J Sports Physiol Perform. (2022) 17(7):995–1010. 10.1123/ijspp.2021-0104 35523423

[49] Lakicevic N Roklicer R Bianco A Mani D Paoli A Trivic T. Effects of rapid weight loss on judo athletes: a systematic review. Nutrients. (2020) 12(5):1220. 10.3390/nu12051220 32357500 PMC7281976

[50] Martínez-Aranda LM Sanz-Matesanz M Orozco-Durán G González-Fernández FT Rodríguez-García L Guadalupe-Grau A. Effects of different rapid weight loss strategies and percentages on performance-related parameters in combat sports: an updated systematic review. Int J Environ Res Public Health. (2023) 20(6):5158. 10.3390/ijerph20065158 PMC10048848 36982067

[51] Reale R Slater G Burke LM. Acute-weight-loss strategies for combat sports and applications to Olympic success. Int J Sports Physiol Perform. (2017) 12(2):142–51. 10.1123/ijspp.2016-0211 27347784

[52] Fullagar HHK Skorski S Duffield R Hammes D Coutts AJ Meyer T. Sleep and athletic performance: the effects of sleep loss on exercise performance, and physiological and cognitive responses to exercise. Sports Med. (2015) 45(2):161–86. 10.1007/s40279-014-0260-0 25315456

[53] Amri A Faleh J Ben Aissa M Ceylan Hİ Ben Hassen S Hmaidi J. Effects of sleep restriction on cognitive and physical performance in elite karate athletes: a randomized crossover study. Medicine (Baltimore). (2026) 105(11):e48064. 10.1097/MD.0000000000048064 41824810 PMC12991690

[54] López-Laval I Mielgo-Ayuso J Terrados N Calleja-González J. Evidence-based post exercise recovery in combat sports: a narrative review. J Sports Med Phys Fitness. (2021) 61(3):386–400. 10.23736/S0022-4707.20.11341-0 33092328

[55] Vickers AJ Elkin EB. Decision curve analysis: a novel method for evaluating prediction models. Med Decis Making. (2006) 26(6):565–74. 10.1177/0272989X06295361 17099194 PMC2577036

[56] Atkinson G Nevill AM. Statistical methods for assessing measurement error (reliability) in variables relevant to sports medicine. Sports Med. (1998) 26(4):217–38. 10.2165/00007256-199826040-00002 9820922

[57] Hopkins WG. Measures of reliability in sports medicine and science. Sports Med. (2000) 30(1):1–15. 10.2165/00007256-200030010-00001 10907753

[58] Morales J Alamo JM García-Massó X Buscà B López JL Serra-Añó P. Use of heart rate variability in monitoring stress and recovery in judo athletes. J Strength Cond Res. (2014) 28(7):1896–905. 10.1519/JSC.0000000000000328 24276307

[59] Moons KGM Kengne AP Woodward M Royston P Vergouwe Y Altman DG. Risk prediction models: I. Development, internal validation, and assessing the incremental value of a new (bio)marker. Heart. (2012) 98(9):683–90. 10.1136/heartjnl-2011-301246 22397945

[60] Moons KGM Kengne AP Grobbee DE Royston P Vergouwe Y Altman DG. Risk prediction models: II. External validation, model updating, and impact assessment. Heart. (2012) 98(9):691–8. 10.1136/heartjnl-2011-301247 22397946

[61] Steyerberg EW Vickers AJ Cook NR Gerds T Gonen M Obuchowski N. Assessing the performance of prediction models: a framework for traditional and novel measures. Epidemiology. (2010) 21(1):128–38. 10.1097/EDE.0b013e3181c30fb2 20010215 PMC3575184

[62] Bullock GS Mylott J Hughes T Nicholson KF Riley RD Collins GS. Just how confident can we be in predicting sports injuries? A systematic review of the methodological conduct and performance of existing musculoskeletal injury prediction models in sport. Sports Med. (2022) 52(10):2469–82. 10.1007/s40279-022-01698-9 35689749

[63] Austin PC Steyerberg EW. Predictive accuracy of risk factors and markers: a simulation study of the effect of novel markers on different performance measures for logistic regression models. Stat Med. (2013) 32(4):661–72. 10.1002/sim.5598 22961910 PMC3575692

[64] Saw AE Main LC Gastin PB. Monitoring the athlete training response: subjective self-reported measures Trump commonly used objective measures: a systematic review. Br J Sports Med. (2016) 50(5):281–91. 10.1136/bjsports-2015-094758 26423706 PMC4789708

[65] Negaresh R Del Coso J Mokhtarzade M Lima-Silva AE Baker JS Willems MET. Effects of different dosages of caffeine administration on wrestling performance during a simulated tournament. Eur J Sport Sci. (2019) 19(4):499–507. 10.1080/17461391.2018.1534990 30378465

[66] Halson SL. Monitoring training load to understand fatigue in athletes. Sports Med. (2014) 44(Suppl. 2):S139–47. 10.1007/s40279-014-0253-z 25200666 PMC4213373

[67] Curran PJ Bauer DJ. The disaggregation of within-person and between-person effects in longitudinal models of change. Annu Rev Psychol. (2011) 62:583–619. 10.1146/annurev.psych.093008.100356 19575624 PMC3059070

[68] Bishop D. An applied research model for the sport sciences. Sports Med. (2008) 38(3):253–63. 10.2165/00007256-200838030-00005 18278985

[69] Williams SJ Kendall L. Perceptions of elite coaches and sports scientists of the research needs for elite coaching practice. J Sports Sci. (2007) 25(14):1577–86. 10.1080/02640410701245550 17852663

[70] Ouergui I Franchini E Selmi O Levitt DE Chtourou H Bouhlel E. Relationship between perceived training load, well-being indices, recovery state and physical enjoyment during judo-specific training. Int J Environ Res Public Health. (2020) 17(20):7400. 10.3390/ijerph17207400 33050671 PMC7650691

[71] Saw AE Main LC Gastin PB. Monitoring athletes through self-report: factors influencing implementation. J Sports Sci Med. (2015) 14(1):137–46. PMID: 25729301. 25729301 PMC4306765

[72] Borg GA. Psychophysical bases of perceived exertion. Med Sci Sports Exercise. (1982) 14(5):377–81. 10.1249/00005768-198205000-00012 7154893

[73] Glazier PS Davids K Bartlett RM. Dynamical systems theory: a relevant framework for performance-oriented sports biomechanics research. Sportscience. (2003) 7:1–8. Available online at: https://sportsci.org/jour/03/psg.htm (Accessed August 25, 2026).

[74] Van Emmerik REA Ducharme SW Amado AC Hamill J. Comparing dynamical systems concepts and techniques for biomechanical analysis. J Sport Health Sci. (2016) 5(1):3–13. 10.1016/j.jshs.2016.01.013 30356938 PMC6191988

